# Variation analysis of six HCV viral load assays using low viremic HCV samples in the range of the clinical decision points for HCV protease inhibitors

**DOI:** 10.1007/s00430-014-0364-z

**Published:** 2014-11-15

**Authors:** F. Wiesmann, G. Naeth, C. Sarrazin, A. Berger, R. Kaiser, R. Ehret, H. Knechten, P. Braun

**Affiliations:** 1 HIV and Hepatitis Research Group, PZB Aachen, Blondelstr. 9, 52062 Aachen, Germany; 2 Medical Clinic 1, Johann Wolfgang Goethe-University, Frankfurt, Germany; 3 Institute of Medical Virology, Johann Wolfgang Goethe-University, Frankfurt, Germany; 4 Institute of Virology, University of Cologne, Cologne, Germany; 5 Medical Laboratories Berg, Berlin, Germany

**Keywords:** HCV, Quantification, Viremia, COBAS, TaqMan, RealTi*m*e

## Abstract

In the range of clinical decision points for response-guided therapy of HCV, there is still insufficient data concerning the conformity of quantification results obtained by different assays and their correlation with the HPS/CTM v2 assay which was used for initial clinical studies. In a head-to-head comparison, assay accuracy and detection rates of six quantitative assays [*artus* HCV QS-RGQ, COBAS Ampliprep/COBAS TaqMan HCV v1/v2, High Pure System/COBAS TaqMan (HPS), RealTi*m*e HCV, and Versant HCV1.0] were assessed by measuring WHO and PEI standards at dilution steps near clinical decision points. Detection rates and mean differences between assays were evaluated by analyzing twenty clinical samples at 10, 100, and 1,000 IU/mL. Ten replicates from specimens with different HCV genotypes were used to analyze pan-genotypic intra-assay variation. At ≤25 IU/mL, RealTi*m*e demonstrated the highest detection rates. With 0.1 log difference when testing clinical samples, results obtained from the Versant and RealTi*m*e assays matched best with results from HPS. Mean difference analysis across all assay results revealed wide differences between 0.01 and 0.75 log IU/mL. RealTi*m*e showed the lowest intra-assay variation across genotypes 1–4 (25, 100, 1,000 IU/mL). There are substantial analytical differences between viral load assays clinicians should be aware of. These variations may have impact on clinical decisions for patients on HCV triple therapy and may argue for assay-specific decision points equivalent to reference values established in studies using HPS. A comparison of quantification is recommended prior to a switch of assays during ongoing therapy.

## Introduction

The treatment of hepatitis C virus infections has been complicated due to low treatment success rates and/or high rates of treatment discontinuation in consequence of side effects. The former standard of care therapy for HCV-infected patients included a combination therapy with interferon and *Ribavirin*, both of which have nonspecific and largely unknown mechanisms of action. The development of directly acting antivirals (DAAs) has further improved new treatment options for patients who had been infected with the, formerly prognostically most unfavorable, genotype 1 [1].

However, those new treatment options may come along with high costs and an increased risk to develop severe treatment side effects. In this context, stopping rules were defined in the lower range of quantification to avoid unnecessary continuation of therapy [2]. For example, treatment with the NS3/4A protease inhibitor (PI) *Boceprevir* should be discontinued at week 12, if HCV RNA is ≥100 IU/mL, or >1,000 at week 4 or 12 when using the HCV protease inhibitor *Telaprevir*, respectively [3]. Triple therapies with the more recently approved protease inhibitor *Simeprevir* require a control of viral load after week 4, with a cutoff of 25 IU/mL, and week 12, where viral load should be undetectable. These stopping rules are based on viral load data generated during the clinical phase II/III studies using the *High* *Pure* *System (HPS)/COBAS TaqMan v2* (Roche) [4–7].

Qiagen *artus* HCV QS-RGQ, Roche COBAS AmpliPrep/COBAS TaqMan HCV versions 1.0 and 2.0, Abbott RealTi*m*e HCV assay, and the Siemens Versant HCV RNA version 1.0 are commercially available automated assays used for the quantification of HCV viral load while the Roche COBAS TaqMan HCV test for use with the High Pure System version 2.0 includes a manual extraction procedure. The assays were calibrated based on different historical WHO standards and vary in sensitivity and quantification range (Table 1).

**Table 1:** Overview of assay characteristics as provided by the manufacturers at date of analysis

	*artus*	CTM v1	CTM v2	HPS	RealTi*m*e	Versant
Manufacturer	Qiagen	Roche	Roche	Roche	Abbott	Siemens
Reference standard	Acrometrix	1st WHO	2nd WHO	2nd WHO	2nd WHO	3rd WHO
Input volume (mL)	1.0	0.85	0.5	0.5	0.5	0.5
Target region	5′-UTR					
LLOQ (IU/mL)	67.6	43	15	25	12	15
LOD (IU/mL)	36.2	15	15	~9.3–16.1	12	15

*LLOQ* lower limit of quantification, *LOD* limit of detection

There is little information available concerning the comparability of results obtained by these six different assays in low viremic HCV samples, and there exists a particular interest to estimate the inter- and intra-assay variation at the lower end of the linear range of each assay, also with respect to different viral genotypes and the impact on patient management.

## Materials and methods

### HCV RNA viral load assays

Six commercially available quantitative HCV RNA assays were evaluated during this investigation: Qiagen *artus* HCV QS-RGQ (*artus*), Roche COBAS AmpliPrep/COBAS TaqMan HCV version 1.0 (CTM v1), Roche COBAS AmpliPrep/COBAS TaqMan HCV version 2.0 (CTM v2), Roche COBAS TaqMan HCV test for use with the High Pure System version 2.0 (HPS), Abbott RealTi*m*e HCV assay (RealTi*m*e), and the Siemens Versant HCV RNA version 1.0 (Versant). Assay characteristics are summarized in Table 1.

### Preanalytics

Serial dilutions of WHO and PEI standards and clinical specimens were prepared using HCV-negative Basematrix (Seracare Life Sciences; HCV negativity was confirmed by using the Abbott RealTi*m*e HCV assay). Afterward, these dilutions were aliquoted according to testing schedule and number of assays investigated. All plasma samples were treated identically and underwent the same number of thawing cycles. Dilutions were prepared using HCV-negative Basematrix as mentioned above.

A total of five laboratories participated in this study. Samples were shipped on dry ice to the respective laboratories, and successful delivery in a frozen state was confirmed.

### Accuracy and detection rates using serially diluted WHO and PEI standards

Serial dilution panels at nominal concentrations of 1,000, 500, 200, 100, 25, 10, and 5 IU/mL were prepared from the 3rd WHO international standard (NIBSC code 06/100, genotype 1a) and the German PEI reference HCV RNA standard (3443/04, Paul-Ehrlich-Institut, Germany, genotype 1a). Triplicates of panel members ranging from 1,000 to 25 IU/mL were tested in a single run across the above-mentioned six different HCV viral load assays. For each panel member, accuracy was determined using the observed median concentrations compared with the nominally assigned values from WHO and PEI.

Detection rates at extremely low viremic levels were assessed by testing triplicates of panel members within the range of 5–25 IU/mL.

### Quantification of 20 clinical HCV genotype 1 samples

Archived leftover plasma specimens from 20 patients infected with HCV genotype 1 were serially diluted to concentrations of 1,000, 100, and 10 IU/mL based on previous routine quantification with RealTi*m*e, respectively. Three replicates of each sample were tested by each of the six HCV viral load assays. The differences between the various assays and especially in comparison with the results of the HPS assay were estimated by mean difference analysis. Differences between quantification results for genotype 1a and 1b samples were investigated by scatter plot analysis, Mann–Whitney *U* test, *t* test after Kolmogorov–Smirnov and Shapiro–Wilk test for normality.

### Quantification and intra-assay variation analysis using low viremic clinical samples of different HCV genotypes

Archived leftover plasma specimens from patients infected with HCV genotype 1a and 1b (*n* = 3 each), and genotype 2, 3, and 4 (*n* = 1 each) were diluted to target concentrations of 1,000, 100, and 25 IU/mL based on previous RealTi*m*e results, respectively. Each sample was analyzed in 10 independent runs using one replicate per run across all six HCV viral load assays.

At each dilution of 1,000, 100, and 25 IU/mL, mean coefficients of variation (CV) were calculated if at least 50 % of the clinical samples had results ≥LLOQ for atleast 50 % of their replicates.

### Range of uncertainty (RoU)

An additional statistical analysis for evaluating a range of uncertainty was based on precision and mean difference data of genotype 1 clinical samples compared with HPS assay results. The calculations for the lower and upper limit of the range of uncertainty were derived from two one-sided confidence limit calculations approaching the clinical decision point either from lower or higher viral loads as suggested in [8]:

1 $${\text{RL}}_{\text{Low}} = \frac{{{\text{CO}}_{\text{A}} }}{{ 1 + \left( {z \times \frac{{{\text{CV\% }}}}{\sqrt n }} \right)}},\quad {\text{RL}}_{\text{Up}} = \frac{{{\text{CO}}_{\text{A}} }}{{ 1 + \left( {z \times \frac{{{\text{CV\% }}}}{\sqrt n }} \right)}},$$

(RL, limits of the range of uncertainty; CO_A_, assay-specific equivalent of the clinical cutoff established by the reference assay; CV%, assay-specific coefficient of variation at the respective cutoff level; *n*, number of replicates). Calculations were assuming a 95 % confidence level resulting in a *z* value of 1.645.

## Results

### Evaluation of assay accuracy and detection rates using PEI and WHO standards

**Fig. 1:**
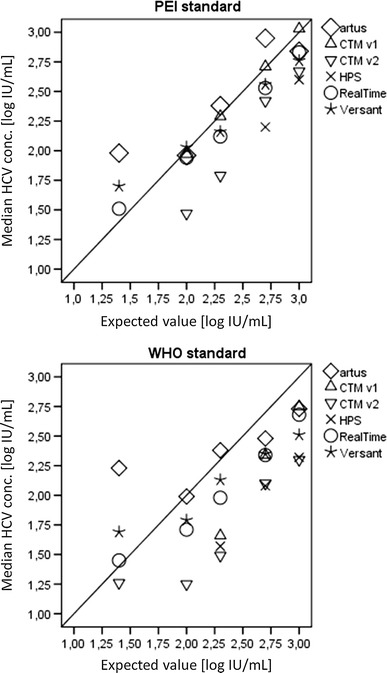
Assay accuracy of all six assays analyzing panels of WHO and PEI standard replicates with nominal concentrations of 25–1,000 IU/mL, respectively

Figure 1 shows the correlation of the six investigated HCV RNA assays with the PEI and WHO standards across a serial dilution panel of 25–1,000 IU/mL nominal standard concentrations. Differences between median assay results and nominal concentrations of the standard panel members are listed in Table 2 and were found to be consistently <0.5 log IU/mL only for RealTi*m*e (maximum 0.36 log IU/mL) and Versant (maximum 0.49 log IU/mL). For the other assays, results differed up to 0.98 log IU/mL (CTM v2). *artus* results showed only small differences from nominal values at concentrations of 100 IU/mL and above but tended to either overestimate concentrations <100 IU/mL or HCV RNA was not detected or quantified. Different to median PEI standard results >100 IU/mL, the corresponding median WHO standard results tended to be quantified at lower values than expected by virtually all assays.

**Table 2:** Overview of absolute and logarithmic PEI/WHO quantification results of all six compared assays with nominal concentrations of 25–1,000 IU/mL

Nominal standard	Median concentrations																	
	*artus*			CTM v1			CTM v2			HPS			RealTi*m*e			Versant		
IU/mL [log IU/mL]	IU/mL	Range	log IU/mL	IU/mL	Range	log IU/mL	IU/mL	Range	log IU/mL	IU/mL	Range	log IU/mL	IU/mL	Range	log IU/mL	IU/mL	Range	log IU/mL
1,000 [3.0] PEI	699	497–884	2.84	1070	890–1110	3.03	468	450–603	2.67	399	236–455	2.60	675	648–718	2.83	575	533–747	2.76
1,000 [3.0] WHO	543	319–627	2.73	568	373–757	2.75	200	166–564	2.30	209	156–299	2.32	481	405–508	2.68	322	321–473	2.51
500 [2.7] PEI	898	797–963	2.95	511	391–557	2.71	263	187–429	2.42	159	150–184	2.20	340	319–364	2.53	359	305–434	2.56
500 [2.7] WHO	303	300–482	2.48	217	161–238	2.34	127	113–473	2.10	120	101–147	2.08	220	214–243	2.34	223	204–249	2.35
200 [2.3] PEI	239	238–345	2.38	193	122–198	2.29	62	51–94	1.79	det.	det.–69	det.	132	127–174	2.12	144	81–148	2.16
200 [2.3] WHO	227	det.–245	2.36	46	44–61	1.66	21	14–46	1.32	29	det.–48	1.46	96	93–106	1.98	125	105–134	2.10
100 [2.0] PEI	91	86–100	1.96	94	78–98	1.97	29	25–41	1.61	det.	det.–34	det.	87	67–93	1.94	106	97–108	2.03
100 [2.0] WHO	92	det.–103	1.96	det.	det.	det.	17	14–19	1.23	det.	det.	det.	51	32–62	1.71	62	57–79	1.79
25 [1.4] PEI	91	det.–98	1.96	det.	det.	det.	det.	det.–21	det.	det.	det.	det.	32	31–41	1.51	50	46–100	1.70
25 [1.4] WHO	150	0–185	2.18	det.	det.	det.	det.	0–18	det.	det.	det.	det.	28	15–33	1.45	49	23–50	1.69

det. = detected but below LLOQ; 0 = target not detected

Detection rates of replicates of the WHO and PEI panel members with nominal concentrations of 25, 10, and 5 IU/mL considerably varied across the assays investigated (Table 3). RealTi*m*e and Versant were able to quantify each of the six replicates of the WHO and PEI panel members at a nominal concentration of 25 IU/mL. In contrast, although detecting virtually all replicates, CTM v2 quantified only two and HPS quantified none out of six. At a concentration of 10 IU/mL, solely, CTM v1 and RealTi*m*e detected HCV RNA in all measured replicates, whereas the other assays did not detect HCV RNA in varying numbers of replicates. RealTi*m*e turned out to be the most sensitive assay compared with the other systems by detecting four out of six PEI and WHO replicates at nominal concentrations of 5 IU/mL as well as by showing the highest overall detection rate (89 %) at nominal concentrations of 5–25 IU/mL, followed by CTM v1 (78 %), HPS (67 %), Versant (56 %), *artus* (50 %), and CTM v2 (39 %).

**Table 3:** Analysis of assay detection rates at 5–25 IU/mL nominal concentration of PEI and WHO replicates

Assay	Number of detected samples (IU/mL)			Overall detection rate (%)
	25	10	5	
RealTi*m*e	6/6	6/6	4/6	89
CTM v1	6/6	6/6	2/6	78
HPS	6/6	4/6	2/6	67
Versant	6/6	3/6	1/6	56
*artus*	5/6	2/6	2/6	50
CTM v2	5/6	1/6	1/6	39

### Quantification of 20 clinical HCV genotype 1 samples

**Fig. 2:**
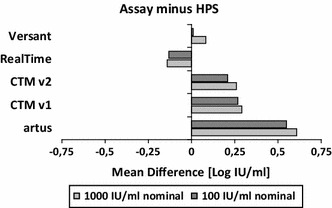
Mean difference analysis in comparison with HPS results (measuring 20 clinical genotype 1 samples at clinical decision points with three replicates each)

**Fig. 3:**
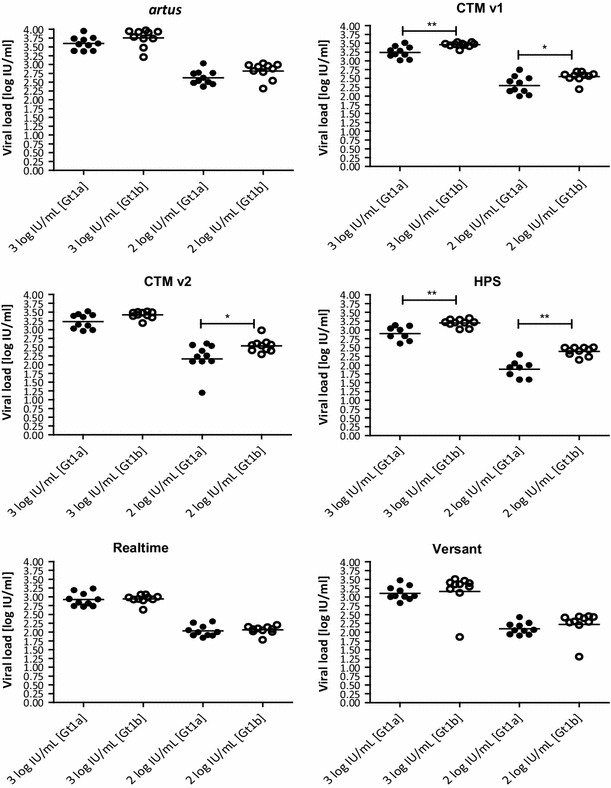
Genotype 1a versus genotype 1b quantification based on means of 10 clinical samples with three replicates each [* or ** results are significantly different (*p* < 0.05) or (*p* < 0.01) as determined by unpaired Mann–Whitney and *t* test after Kolmogorov–Smirnov and Shapiro–Wilk test for normality]

On the basis of 20 clinical HCV genotype 1 samples [genotype 1a (*n* = 10); genotype 1b (*n* = 10)], measured in triplicates, the extent of assay variation at the clinical decision points 100 and 1,000 IU/mL was demonstrated by mean difference analysis (Fig. 2). HPS, which was used as reference assay for establishing clinical decision points concerning stop or continuation of HCV treatment in clinical trials, showed the highest overall concordance with results obtained by RealTi*m*e or Versant with differences <0.15 log IU/mL (Fig. 2; Table 4). Table 4 provides an overview of the quantification differences across all assays which ranged from 0.01 to 0.75 log IU/mL. With a difference of 0.29–0.75 log IU/mL, for instance, mean *artus* results tended to be measured clearly above the results found by other assays independent of the dilution analyzed. CTM v1 and CTM v2 showed only marginal discrepancies between each other but differed more than 0.2 log IU/mL from mean results measured by *artus*, RealTi*m*e, or HPS. An additional subanalysis identified statistically significantly higher concentrations for genotype 1b samples compared with genotype 1a samples when being analyzed with HPS, CTM v1, or CTM v2 (Fig. 3).

**Table 4:** Mean difference analysis across all six investigated assays measuring 20 clinical genotype 1 samples (three replicates each) at clinical decision points 1000 IU/mL (left columns) and 100 IU/mL (right columns per assay)

Assay 1 minus assay 2	Assay 2											
Mean difference [log IU/mL]	*artus*		CTM v1		CTM v2		HPS		RealTi*m*e		Versant	
Assay 1												
*artus*	–		0.33	0.29	0.35	0.35	0.61	0.55	0.75	0.68	0.53	0.55
CTM v1	−0.33	−0.29	–		0.02	0.06	0.29	0.27	0.42	0.38	0.20	0.26
CTM v2	−0.35	−0.35	−0.02	−0.06	–		0.26	0.21	0.40	0.33	0.17	0.20
HPS	−0.61	−0.55	−0.29	−0.27	−0.26	−0.21	–		0.14	0.13	−0.08	−0.01
RealTi*m*e	−0.75	−0.68	−0.42	−0.38	−0.40	−0.33	−0.14	−0.13	–		−0.22	−0.13
Versant	−0.53	−0.55	−0.20	−0.26	−0.17	−0.20	0.08	0.01	0.22	0.13	–	

Mean differences determined by calculating mean value of assay 1 minus mean value of assay 2

In a further analysis, the ability of each assay to detect clinical samples at nominal HCV RNA concentrations of 10 IU/mL was tested on 60 replicates derived from the same 20 clinical samples (Table 5). CTM v2, RealTi*m*e, and Versant were able to detect all of the analyzed specimens. Two replicates were not detected by CTM v1 and HPS, respectively; *artus* missed ten replicates.

**Table 5:** Ability to detect HCV RNA in 20 clinical genotype 1 samples at 10 IU/mL (three replicates each)

System	*N*	Not detected	Positive < LLOQ	≥LLOQ
*artus*	60	10	22	28
CTM v1	58*	2	54	2
CTM v2	59*	0	23	36
HPS	54**	2	47	5
RealTi*m*e	60	0	30	30
Versant	60	0	22	38

* One or two replicates missing due to technical issues

** Six replicates missing due to lack of sample material

### Quantification and analysis of assay variation within each assay by using low viremic clinical samples of different HCV genotypes

**Fig. 4:**
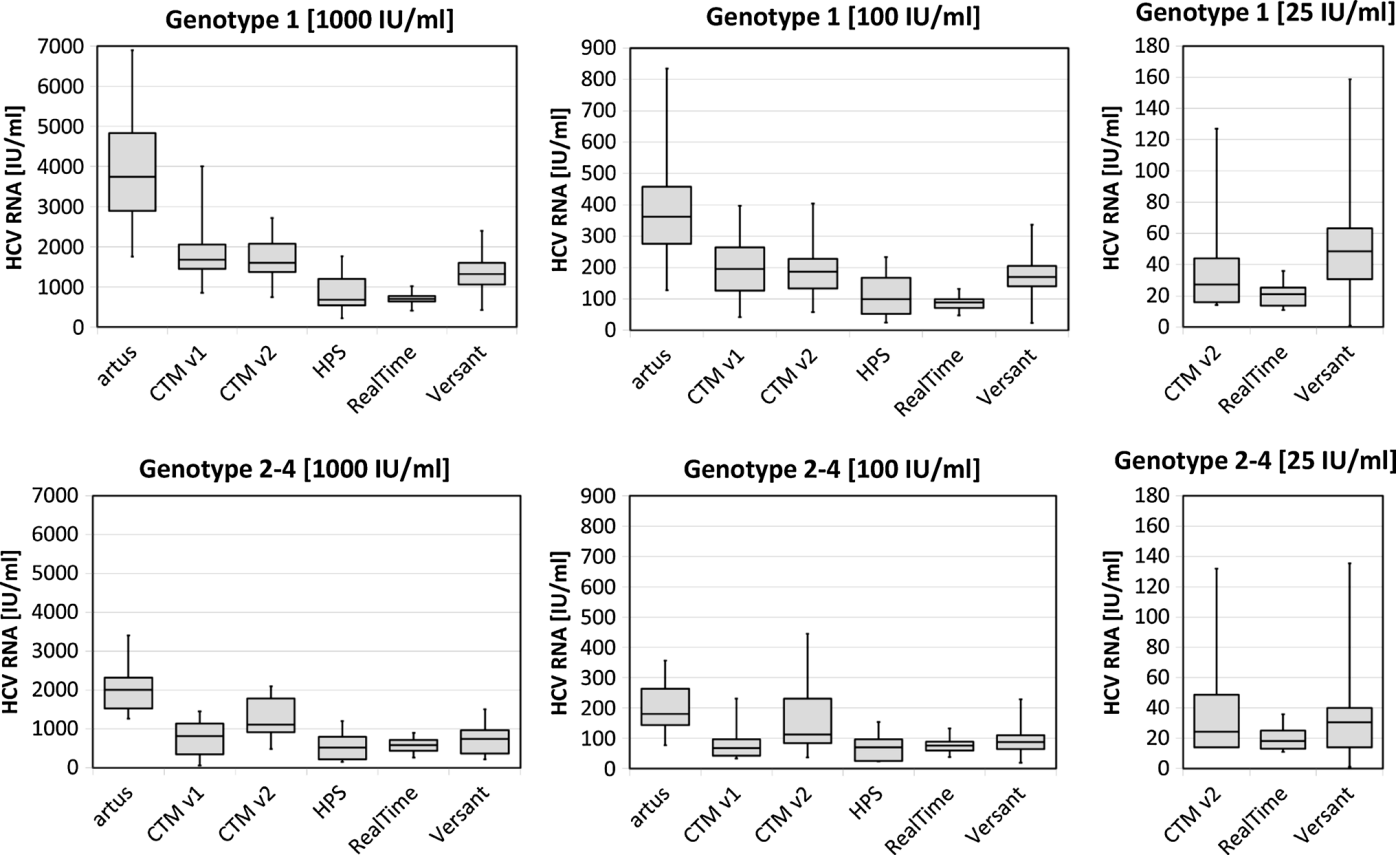
Intra-assay variations for absolute values as illustrated in *boxplot* analysis (*n* = 3 samples for each of both subtypes 1a and 1b, *n* = 1 sample for genotypes 2–4, respectively; in total ten replicates per sample measured in 10 independent runs; no *boxplots* for *artus*, CTM v1, and HPS at 25 IU/mL due to insufficient number of quantified replicates)

**Fig. 5:**
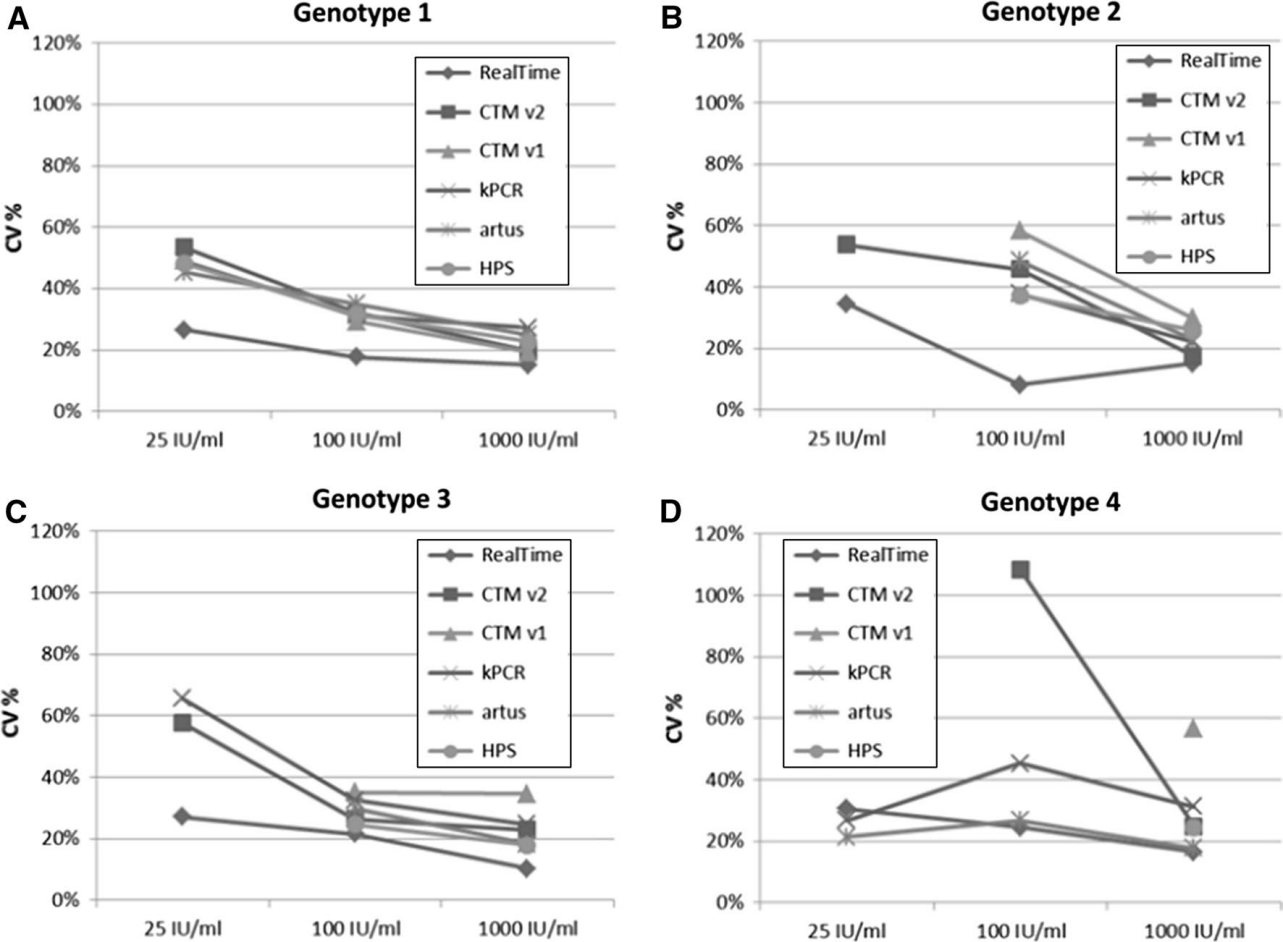
Mean coefficients of variation for ten replicates from 25 to 1,000 IU/mL nominal concentration and genotypes 1 (**a**), 2 (**b**), 3 (**c**), and 4 (**d**)

Figure 4 demonstrates the intra-assay variations for different concentrations of the HCV genotypes 1, 2, 3, and 4. Samples were tested in ten independent runs using one replicate of each sample per run across the six different HCV assays, respectively. CV% values across the tested genotypes and across the nominal concentrations 1,000, 100, and 25 IU/mL ranged between 8 and 34 % for RealTi*m*e, 18–48 % for *artus*, 19–58 % for CTM v1, 17–108 % for CTM v2, 18–48 % for HPS, and 22–65 % for Versant (Fig. 5). Overall, RealTi*m*e showed the highest precision across all target concentrations and across genotypes 1, 2, and 3. For genotype 4, CV values obtained with *artus* and partially Versant were in a comparable low range as for RealTi*m*e (Fig. 5).

The less sensitive assays *artus*, CTM v1, and HPS failed to quantify the majority or even all replicates at 25 IU/mL across all tested genotypes 1–4. Additionally, Versant failed in quantifying most of genotype 2 replicates at 25 IU/mL. The majority of genotype 4 replicates were furthermore neither quantified by CTM v2 at 25 IU/mL nor by CTM v1 and HPS at 100 IU/mL. Thus, RealTi*m*e was the only assay that quantified the majority of replicates at target concentrations of 25 IU/mL across all genotypes 1–4.

The differences in quantification across the assays as already shown in Fig. 2 were confirmed by these results (Fig. 4).

### Range of uncertainty (RoU)

**Fig. 6:**
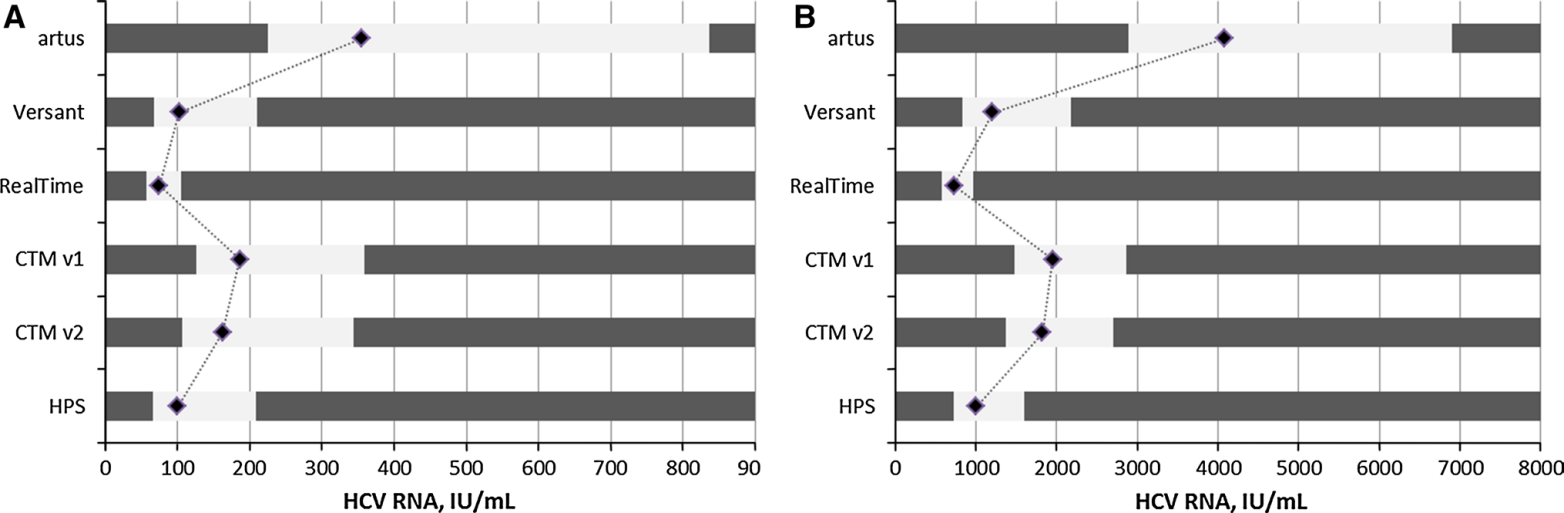
Range of uncertainty for different assays in relation to HPS at 100 or 1,000 IU/mL [8]. *Black bars* represent areas of ≥95 % confidence to not cross the decision threshold of the 618 100 IU/mL (**a**) or 1,000 IU/mL (**b**) HPS value analogues. *Diamonds* refer to the assay-specific HPS 619 equivalents. *Gray bars* refer to the RoU

The RoUs for the different assays in relation to the stopping rules evaluated with the HPS system are shown in Fig. 6 based on the data in Table 6. The reference points (indicated by black diamonds) refer to the equivalent HPS result considering assay-specific mean differences to HPS. The RoU exhibits assay-specific imprecision, differences in quantification compared with the assay used for evaluating the rules, and confidence limits for reporting results and guiding appropriate decisions [8]. Values beyond the upper and below the lower limits of the RoU refer to the values in relation to the HPS reference that provide at least 95 % confidence not to cross the clinical decision point. Consequently, test results within the RoU do not provide sufficient confidence to guide a decision and might be subject to further evaluation [9].

**Table 6:** Range of uncertainty characteristics

	100 IU/mL			1,000 IU/mL		
	Ref. value	LL	UL	Ref. value	LL	UL
*artus*	355	225	836	4,074	2,890	6,900
Versant	102	68	210	1,202	830	2,182
RealTi*m*e	74	57	105	724	580	964
CTM v1	186	126	358	1,950	1,480	2,857
CTM v2	162	106	344	1,820	1,373	2,699
HPS	100	66	208	1,000	729	1,592

*LL* lower limit, *UL* upper limit; all numbers in IU/mL

## Discussion

Assay precision and accuracy are of great clinical relevance since only one single measurement is used to predict whether a patient will or will not achieve SVR and therefore is eligible to continue an expensive antiviral therapy. Precision and accuracy especially in the low viremic range should be assured in order to properly determine viral loads. Furthermore, assay sensitivity plays a role in RGT since patients with undetectable HCV RNA at certain time points may be assigned to a shortened antiviral therapy.

Unfortunately, the above-mentioned clinical decision points were established using HPS, a manual assay that is rarely used in daily routine. Differences in HCV RNA quantification compared with HPS need to be evaluated in order to avoid inappropriate discontinuation of therapy or unnecessarily prolonged treatment.

In this analysis, assay accuracy was investigated using a serial dilution panel prepared from PEI and WHO standards at a nominal concentration range of 25–1,000 IU/mL. The assay results vary in the extent of deviation from the expected values. Potential contributing factors could be the adjustment of the assays to different reference standards but also differences in assay designs. Overall, the relationship between expected and measured values for the individual assay is consistent across both PEI and WHO standard panels although the measured values at higher concentration levels are generally lower for WHO than for PEI standard replicates.

In addition, detection rates using low concentration members of the WHO and PEI panels (25, 10, 5 IU/mL) were compared across the assays. RealTi*m*e showed the highest overall detection rate of 89 % in this analysis, followed by CTM v1 and HPS with 78 and 67 %, respectively. The lowest overall detection rate of 39 % was found using CTM v2. In contrast, when evaluating sensitivity in 20 clinical genotype 1 samples at a target dilution of 10 IU/mL, again RealTi*m*e but also CTM v2 and Versant showed 100 % detection rates, while detection rates for *artus*, CTM v1, and HPS were lower (83, 97, and 96 %, respectively).

A recent publication by [10] likewise revealed a limited assay concordance of only 55 % comparing sensitivities of HPS, CTM v1, and CTM v2 in terms of detecting low HCV viremia at week 4 in RGT [10]. Similar differences between various assays were also found in other studies [11–13]. In 2013, Ogawa et al. [14] reported that despite undetectable HCV RNA in week 4 by CTM v1 indicating rapid virologic response (RVR), three patients (6.2 %) failed to achieve SVR. However, all patients with RVR according to the RealTi*m*e results achieved SVR. Furthermore in 2013, Sarrazin et al. [15] presented data supporting a RealTi*m*e-specific clinical cutoff of <12 IU/mL being equivalent to ‘undetected’ results by HPS for decisions on therapy truncation.

Differences in assay quantification were investigated by testing 20 clinical genotype 1 samples in triplicate at clinical decision points (target concentrations of 1,000 and 100 IU/mL). Versant and RealTi*m*e results matched best with the results obtained by HPS, the assay used to establish clinical decision points in clinical studies with protease inhibitors. Mean difference analysis of the results of all assays revealed quantification differences between 0.01 and 0.75 log IU/mL. Remarkably, throughout the entire study, *artus* measured the highest mean concentrations in the clinical samples with the overall largest viral load difference (0.75 log IU/mL) being observed between *artus* and RealTi*m*e. This confirms similar results found by Drexler et al. [16]. Mean differences around 0.3–0.4 log IU/mL between RealTi*m*e and CTM v1 or CTM v2 confirm a previously reported trend [17–19]. Differences between genotype 1a and 1b quantification as obtained by HPS and CTM in this investigation were also observed by LaRue et al. [20] in 2012 for CTM v1 though without statistical significance. Any impact on clinical decisions or other relevance due to the fact that all assays are standardized against genotype 1a standards [21, 22] may be addressed in future studies.

The comparison of six commercially available HCV RNA assays revealed substantial differences in assay precision at low viremic levels (25–1,000 IU/mL). RealTi*m*e demonstrated highest precision across all genotypes 1–4, which turned out to be superior compared with the other assays in genotype 1, 2, and 3 replicates (Figs. 4, 5). This was especially apparent at lower viral loads <100 IU/mL. Only for genotype 4, *artus* and partially Versant showed comparable low CV values.

The observed insufficient capability of Versant to quantify the lowest concentration sample of genotype 2 and likewise of CTM v1, CTM v2, and HPS concerning the lower concentrations of genotype 4 samples is in line with previous reports of lower quantification of these genotypes by the respective assays [17, 19, 23, 24]. However, since only a single sample was tested for each of the genotypes 2, 3, and 4, further investigation is needed.

As depicted above, viral load assays applied in daily routine measurements may considerably vary in terms of precision, quantification level, and detection rate. Several suggestions have been made to provide information about measurement uncertainties. The total analytic error (TAE) concepts describe important and well-regarded approaches combining random error, systematic error, and operational specifications. However, with regard to response-guided therapies, the bias of a laboratory result from a true value may not be as relevant as its relation to a clinical decision point. Therefore, we discussed our findings with the range of uncertainty concept (RoU) that specifically provides confidence information with regard to clinical decision points evaluated by reference methods. Recently, the RoU concept has been applied to HCV stopping rules [8], and here, we tested it for the first time using the results from a head-to-head comparison study.

The RoU illustrates and compares assay-specific imprecision and relative quantification differences compared to a reference, here HPS, in a single chart (Fig. 6). It also provides information on the confidence of a test result in relation to a cutoff evaluated with a different reference assay (HPS). The RoU estimates a laboratory’s specific probability of supporting appropriate decisions and mitigating the risk of inappropriate decisions. Assay-specific clinical decision points equivalent to the HPS reference values as indicated in Fig. 6 and retesting to reduce large RoUs may be considered to improve result reliability.

## Conclusions

Evaluation and comparison of six commercially available HCV RNA assays revealed major differences in assay precision, quantification level, and detection rates. These may impact clinical decisions in particular related to stopping rules in response-guided therapy. Therefore, a comparison of quantification is recommended prior to a switch of assays during ongoing therapy. Thresholds were established by HPS which is rarely used in clinical practice, and there is little information available concerning concordance between HPS and other assays. In this analysis, HPS matched best with results generated by RealTi*m*e and Versant. Moreover, RealTi*m*e combined highest sensitivity with the highest precision across all analyzed genotypes expressed by the lowest mean coefficient of variation. This is of importance since treatment decisions in triple therapy are based on single measurements.

Emerging therapies with compounds like *Simeprevir* and *Daclatasvir* will still include stopping rules using cutoffs at 25 IU/mL [25, 26]. Consequently, assays with reliable precision, accuracy, and sensitivity for quantification of HCV RNA in low level viremia will remain crucial to support optimal clinical decisions in HCV patient management.
